# Exceptionally Long-Lived Individuals (ELLI) Demonstrate Slower Aging Rate Calculated by DNA Methylation Clocks as Possible Modulators for Healthy Longevity

**DOI:** 10.3390/ijms21020615

**Published:** 2020-01-17

**Authors:** Danielle Gutman, Elina Rivkin, Almog Fadida, Lital Sharvit, Vered Hermush, Elad Rubin, Dani Kirshner, Irina Sabin, Tzvi Dwolatzky, Gil Atzmon

**Affiliations:** 1 Department of Human Biology, Faculty of Natural Sciences, University of Haifa, Haifa 3498838, Israel; dgutman546@gmail.com (D.G.); lsharvit@univ.haifa.ac.il (L.S.); 2 Faculty of Public Health, University of Haifa, Haifa 3498838, Israel; duelina2705@gmail.com (E.R.); almogfadida@gmail.com (A.F.); 3 Department of Geriatrics and Skilled Nursing, Laniado Medical Center, Netanya 4244916, Israel; vhermush@laniado.org.il; 4 Ruth and Bruce Rappaport Faculty of Medicine, Technion–Israel Institute of Technology, Haifa 3200003, Israel; kirshner.dani@gmail.com (D.K.); i_sabin@rambam.health.gov.il (I.S.); t_dwolatzky@rambam.health.gov.il (T.D.); 5 Department of Geriatrics, Rambam Health Care Campus, Haifa 3109601, Israel; eladrob@gmail.com; 6 Departments of Genetics and Medicine, Division of endocrinology, Institute for Aging Research and the Diabetes Research Center, Albert Einstein College of Medicine, Bronx, New York, NY 10461, USA

**Keywords:** healthy aging, DNA methylation, epigenetic clocks, telomere length, centenarians

## Abstract

Exceptionally long-lived individuals (ELLI) who are the focus of many healthy longevity studies around the globe are now being studied in Israel. The Israeli Multi-Ethnic Centenarian Study (IMECS) cohort is utilized here for assessment of various DNA methylation clocks. Thorough phenotypic characterization and whole blood samples were obtained from ELLI, offspring of ELLI, and controls aged 53–87 with no familial exceptional longevity. DNA methylation was assessed using Illumina MethylationEPIC Beadchip and applied to DNAm age online tool for age and telomere length predictions. Relative telomere length was assessed using qPCR T/S (Telomere/Single copy gene) ratios. ELLI demonstrated juvenile performance in DNAm age clocks and overall methylation measurement, with preserved cognition and relative telomere length. Our findings suggest a favorable DNA methylation profile in ELLI enabling a slower rate of aging in those individuals in comparison to controls. It is possible that DNA methylation is a key modulator of the rate of aging and thus the ELLI DNAm profile promotes healthy longevity.

## 1. Introduction

Healthy aging is usually characterized by preserved cognitive and motor functions. A unique group of aging individuals termed centenarians serves as a healthy aging model, outliving the age of 100, with mostly intact cognition and physical health [1,2,3]. Such exceptionally long-lived individuals (ELLI) are the focus of many studies around the world [4,5,6,7,8,9,10,11,12], and this group is now being studied in Israel as well. Our newly established cohort of ELLI is part of the Israeli Multi-Ethnic Centenarian Study (IMECS), which aims to elucidate the mechanisms of their healthy aging process. 

Two of the most-studied hallmarks of aging [13] are DNA methylation and telomere attrition. Telomere shortening has long been documented to have inverse correlation with age [14,15,16,17], with mean telomere length (TL) considered a marker for cellular senescence and aging [18,19,20]. Alongside this inverse correlation, mean TL has also been strongly correlated with several age-associated diseases [21,22,23,24,25], adding significance to the negative outcomes of telomere shortening. That said, longer TL has been associated with exceptional longevity [2,26] through several potential mechanisms [27]. Telomere length is commonly measured by southern blot or by quantitative PCR. The latter method has gained popularity for its ease of use and robustness [2,28,29,30,31,32,33,34].

The other hallmark of aging, DNA methylation, increases with age, mostly through a phenomenon termed epigenetic drift [35]. The DNA methylation of centenarians, however, seems to be slightly lower, hinting at a mechanism promoting healthy aging. A study performed on semi-supercentenarians (ages 105–109 years) and their offspring demonstrated that the semi-supercentenarians and their offspring displayed younger “epigenetic age” (calculated on DNA methylation values) with age-matched controls (to the offspring) displaying same “epigenetic age” as actual age [36]. There are several such “epigenetic age” estimators which are mostly developed and utilized using standardized DNA methylation data [35,37,38,39,40,41]. There are various methods for measuring DNA methylation, with the most recently developed Illumina MethylationEPIC beadchip array serving as a thorough, genome-wide, standardized method. Recently, Lu et al. developed two clocks, one for telomere length and one for age, based on DNA methylation levels measured using the Illumina arrays [42,43]. To this date, the DNAmTL or DNAmGrimAge have not been used on DNA methylation data of ELLI. DNAmTL uses 140 CpG sites to estimate telomere length in Kb, while DNAmGrimAge uses 12 sub-DNAm-measures, alongside age and gender, to estimate physiological age with an addition of an estimate of time-to-death termed DNAmAccelGrim. Prior to the development of DNAmGrimAge, the same team developed DNAmPhenoAge, a DNA methylation-based aging biomarker that utilizes 513 CpGs to predict the phenotypic age of an individual [44]. 

The current study utilizes the IMECS cohort (consisting of ELLI, offspring of ELLI, and controls aged 53–87 with no familial exceptional longevity) to compare between the different DNAm clocks and actual phenotypic measures (such as relative telomere length measurements, cognitive performance, and actual age) from the IMECS cohort. We hypothesize that the DNAm age biomarkers and molecular phenotype of ELLI do not differ from those measures in the much younger offspring and control populations. These efforts aim to add knowledge on the phenotype of exceptional longevity and perhaps point at potential therapeutic avenues that might aid in cognitive and physical health preservation or even improvement (as suggested by Fahy et al. [45]).

## 2. Results

### 2.1. DNA Methylation

**Figure 1:**
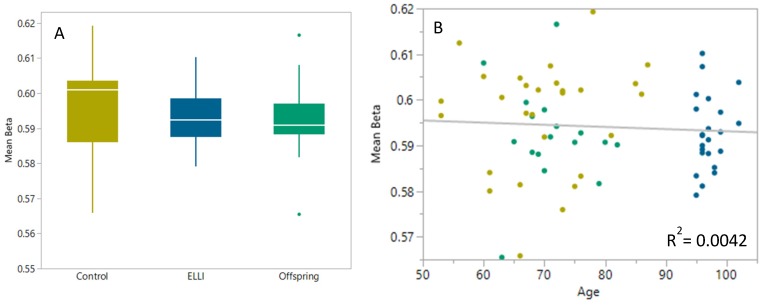
Mean methylation by group. Methylation levels measured via Illumina MethylationEPIC beadchip, converted to beta values with preprocessNoob to remove background read signal through Minfi R package. N_control_ = 28, N_ELLI_ = 24, and N_offspring_ = 17 (one outlier removed). Difference between groups found non-significant by one-way Kruskal–Wallis analysis. (**A**) Mean beta values per group, mean_control_ = 0.596 ± 0.012, mean_ELLI_ = 0.593 ± 0.008, and mean_offspring_ = 0.592 ± 0.011. (**B**) Linear regression of mean beta values over age, non-significant.

DNA methylation raw data of all 70 IMECS participants (described in Table A1 and Table A2) were normalized using Noob normalization, and beta values of all CpG sites passing QC filtering were used to calculate mean beta for each sample, as a measure for global DNA methylation. As can be seen in Figure 1A, the mean beta value for the centenarian group is slightly lower than that of the control group, however this difference was not significant. Lack of significance in this value surprisingly shows great similarity in whole-genome methylation percentage between the groups, hinting at a juvenile methylation profile for ELLI, seeing as global DNA methylation is known to increase with age [35,46]. This similarity is continued, as expected, in the offspring group, demonstrating slightly lower average beta value compared to control as well. In Figure 1B the decrease in methylation with age is easily visible, and contradicting the increase reported by Hannum et al. [35].

### 2.2. DNAm Age Clocks

**Figure 2:**
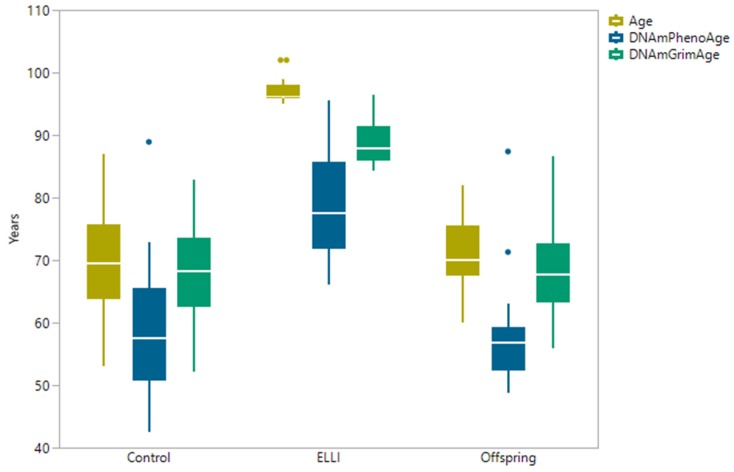
Comparison of actual age and two age clocks. DNAmGrimAge and DNAmPhenoAge calculated by applying methylation beta values to DNAm online tool [37]. N_control_ = 28, N_ELLI_ = 24, and N_offspring_ = 18.

We used two recently developed epigenetic clocks, DNAmPhenoAge [44] and DNAmGrimAge [42], both developed by the Horvath group at UCLA. In short, DNAmPhenoAge is an age clock based on beta values of 513 CpG sites established via correlation with clinical markers, and DNAmGrimAge, the more recent and accurate clock, relies on 12 different sub-DNAm-estimators alongside actual age and gender. Both clocks aim to describe health and lifespan predictions through clinical and phenotypic measurements. Both clocks predict younger age of our groups (Figure 2), with DNAmGrimAge outperforming DNAmPhenoAge, and the ELLI estimations are the most juvenile (differences between actual age and clocks is largest). The differences between chronological age and DNAmGrimAge in the control and offspring groups were very slight (Table A3 and Table A4), whereas the DNAmPhenoAge consistently underestimated the ages of control and offspring participants. This performance is consistent with the DNAmGrimAge performance in the validation data used by the developers, yet is the first to be reported in ELLI, whose ages were calculated to be younger by DNAmGrimAge. 

**Figure 3:**
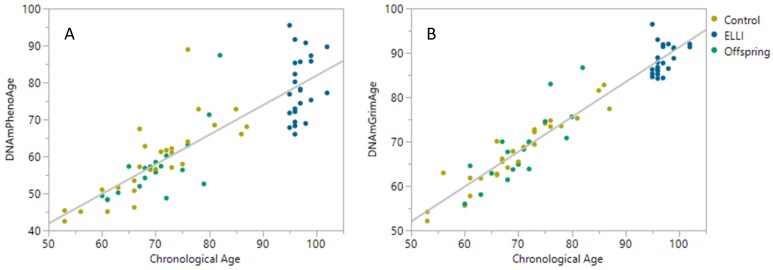
Chronological age vs. DNAm epigenetic age clocks. N_control_ = 28, N_ELLI_ = 24, and N_offspring_ = 18. (**A**) DNAmPhenoAge as a function of chronological age, R^2^ = 0.716, *p* < 0.001. (**B**) DNAmGrimAge as a function of chronological age, R^2^ = 0.919, *p* < 0.001. Linear regressions performed and plotted using JMP 14 (SAS Institute Inc., Cary, NC, USA).

**Figure 4:**
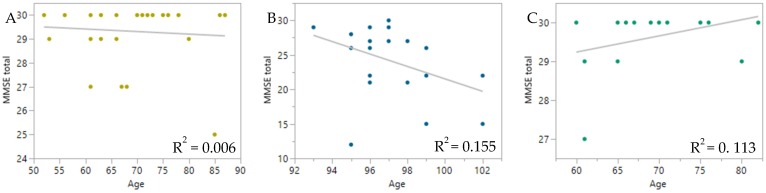
Cognition vs. age. Mini-Mental State Exam scores used for measuring cognitive impairment. (**A**) MMSE score of controls as a function of chronological age, *N* = 28, *p* = 0.7021. (**B**) MMSE score of ELLI as a function of chronological age, *N* = 21, *p* = 0.0776. (**C**) MMSE score of offspring as a function of chronological age, *N* = 16, *p* = 0.2021.

Further, there is a high correlation between chronological age and both DNAm clocks (Figure 3), with DNAmGrimAge outperforming DNAmPhenoAge in actual age prediction. Though DNAmGrimAge is more closely related to chronological age (especially due to the use of actual age as a parameter of DNAmGrimAge), DNAmPhenoAge was originally designed to capture a phenotypic age (rather than chronological age). As depicted by our results, the phenotypic age prediction was lower than the chronological age especially for ELLI, indicating a juvenile phenotype of this group. With this high correlation in mind, we proceeded to examine correlation between age and DNAm clocks with cognitive state of IMECS participants. For this extent, we used the Mini-Mental State Exam (MMSE) questionnaire score as a measure for the cognitive impairment of participants. This assessment revealed no significant correlation between MMSE score and age in neither group (Figure 4). 

### 2.3. Telomere length

**Figure 5:**
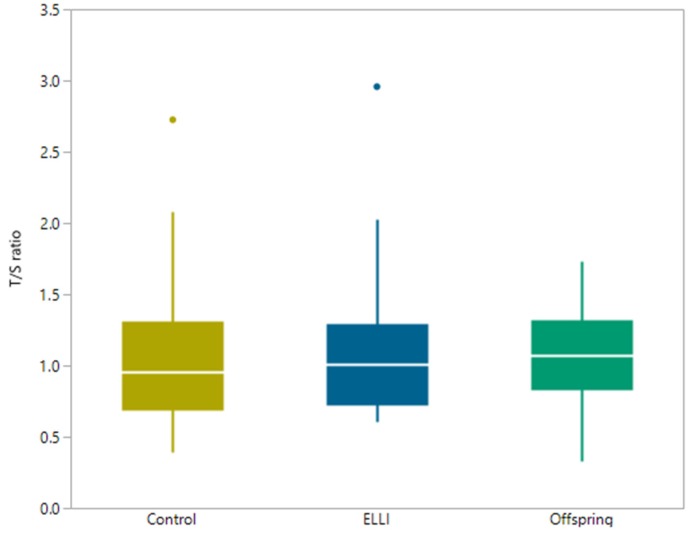
Average T/S ratio as measured by qPCR. T/S ratio obtained by dividing concentration of telomeric reaction by concentration of SCG (Single Copy Gene) reaction, as calculated using standard curve reactions. N_ELLI_ = 12, N_control_ = 17, and N_offspring_ = 12. All pair comparison (Dunn Joint Ranking) non-significant.

**Figure 6:**
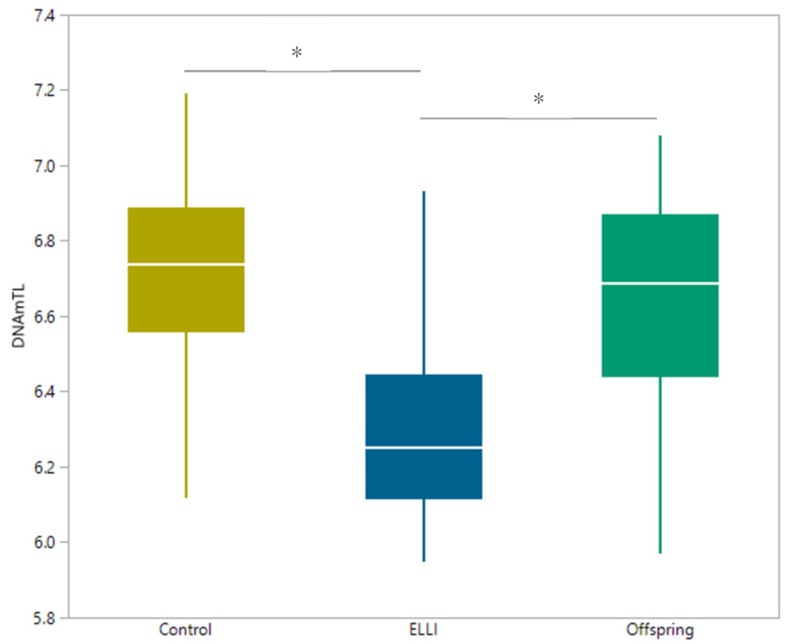
DNAmTL calculated using the online tool. DNAmTL was calculated by applying methylation beta values of 140 CpGs to online tool [37]. N_control_ = 28, N_ELLI_ = 24, and N_offspring_ = 18. * Significant differences, *p* <0.05.

**Figure A1:**
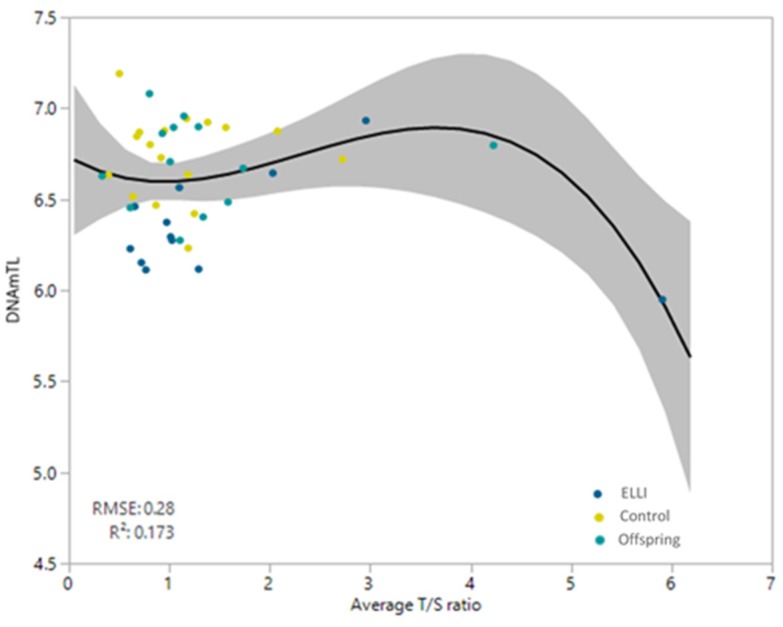
T/S ratio vs. DNAmTL. DNAmTL as a function of T/S ratio (telomeric concentration divided by SCG concentration).

Finally, we turned to telomere length measurement using qPCR and the DNAm estimator of telomere length, DNAmTL. Our qPCR results did not demonstrate different T/S ratios between the three groups (Figure 5 and Table A5). However, the DNAmTL estimator found the telomeres of ELLI to be approximately 500 bp shorter compared to the control and offspring groups (Figure 6 and Table A6). When comparing T/S ratio and DNAmTL (Figure A1), there is no correlation between the two TL measures. Interestingly, when T/S ratio is tested between ELLI and controls with adjustment by DNAmGrimAge, it approaches significant correlation (*p* = 0.0508), hinting at a masking effect of the physiological age (representing juvenile methylation levels of centenarians) on the T/S ratio obtained with qPCR. 

## 3. Discussion

Many studies are aimed at biomarker discovery and improvement for aging [38,41,42,44,47,48,49,50,51,52]. The need for such characterization is of upmost importance in light of efforts to achieve longer health and lifespans across the world. Such biomarker detection would enable tracking and even reversal [45] of aging processes and allow for drug targeting and development to benefit the already graying population. Molecular and genomic biomarkers for aging are still sparse and inaccurate with the exception of the very recent development of DNAmGrimAge [42]. This DNA methylation biomarker outperforms all previously reported methylation age estimators and serves as a very accurate estimate of chronological age. Although this is expected due to the use of chronological as a surrogate for the age prediction, DNAmGrimAge, as DNAmPhenoAge, also serve as an evaluation of health status, indicative of the rate of epigenetic aging. Use of such biomarkers as indication of rate of age acceleration could promote better understanding of the processes underlying progression of aging and replace use of chronological age in clinical assessments relating to those conditions.

That said, the centenarian DNAm still remains elusive, even to the most accurate DNAmGrimAge. We show here that although accurate in offspring of ELLI and unrelated controls, DNAmGrimAge, along with DNAmPhenoAge, underestimates the chronological age of our IMECS ELLI participants, predicting a younger epigenetic age. We believe that this represents a slower rate of aging processes occurring in ELLI, and enabling them to reach such exceptional chronological age. This is in agreement with the methylation profile of semi-supercentenarians and their offspring, described by Horvath et al. [36], and replicates their results in our independent cohort.

The juvenile DNAm profile demonstrated in our cohort together with mostly intact cognition add support to the idea that ELLI age at a slower rate. Even though there was a small decline in the MMSE scores of the ELLI, this decline was not statistically significant, indicating intact cognition in the majority of the ELLI participants.

Further, DNAmTL estimated telomere length compared to T/S ratio of qPCR measurement showed no correlation with each other, until adjusted by DNAmGrimAge, at which the correlation approached significance. This masking effect of the physiological age (measured by DNAmGrimAge) adds support to the slower rate of aging. Telomere length has long been argued for and against use as an age indicator, but it is well-established to be decreased with age. Our qPCR measurements are consistent with previous observations of longer telomeres in ELLI [2]. While T/S ratio of the ELLI was expected to shorten in respect to offspring and controls because of their relatively advanced age, it remained unchanged, indicating a similar telomere length despite almost 30 years average age difference between group participants, demonstrating once again, a decreased aging rate. Taken together with the juvenile methylation rates in ELLI, we suggest that ELLI age slower than the general population through a beneficial methylation profile that may affect telomere length and other aspects of the hallmarks of aging. 

To further draw conclusions, there is a need for bigger sample size and thorough molecular validation. We acknowledge that these are limitations in our current study and are already planning to pursue various directions for validation of our results. In addition, since the work presented here is part of an ongoing study, new IMECS participants are recruited and new recruitment centers should be established to increase ease and rate of recruitment. We believe that with adequate sample size and further validation in primary cells from participants we will be able to obtain more information on the juvenile epigenetic profile of ELLI and their offspring. 

## 4. Materials and Methods 

### 4.1. Ethics Statement

All IMECS participants gave their informed consent for inclusion prior to participation and blood collection. The study was conducted in accordance with the Declaration of Helsinki, and the protocol was approved by the Ethics Committee of Clinical Trials Department, Ministry of Health, Israel (project 109-2014) and by the Institutional Review Board at the Rambam Health Care Center in Haifa, Israel (project RMB 0312-14). Any person over 95 years of age was included in the study with the exclusion of persons cognitively unable to sign informed consent and cognitively impaired persons with no legal guardian. For the offspring group, people with one or more parent outliving the age of 95, either alive or deceased at the time of recruitment, and cognitively able to sign informed consent were included. For the control group any person in the range of 50–90 years of age both of whose parents did not reach 95 years of age and cognitively able to sign informed consent were included.

### 4.2. Sample Collection and Preparation

All IMECS participants (for demographic information see Table A1 and Table A2) underwent physical and cognitive assessment including family history, general health questions, functional assessment including Instrumental Activities of Daily Living (IADL) and Basic Activities of Daily Living (BADL), Mini-Mental State Examination (MMSE), and the 12-item Short Form Health Survey (SF-12). Following this assessment, 20 mL whole blood was drawn from each participant. White blood cells were separated using lymphocyte separation medium (LSM, MP Biomedicals, LLC, CA, USA) and DNA extracted using High Pure PCR Template Preparation Kit (Roche Diagnostics GmbH, Mannheim, Germany) according to manufacturer instructions and quantified using dsDNA High Sensitivity Kit for Qubit (Life technologies Corporation, Eugene, OR, USA) and Qubit (Life Technologies Corporation, Carlsbad, CA, USA). 

### 4.3. DNA Methylation Analysis

For detection of DNA methylation, 500 ng DNA were subjected to bisulfite sequencing and hybridized to Illumina MethylationEPIC beadchip at the Technion Biomedical Core Facilities, Rappaport Faculty of Medicine, Haifa, Israel. Raw data underwent an analysis pipeline using Minfi [53] and BumpHunter [54] (Bioconductor R packages) for quality control and statistical analyses. PreprocessNoob was used for background correction through dye-bias normalization. DNAmTL and all age estimators were obtained using the online tool developed by Lu et al. (https://dnamage.genetics.ucla.edu/home) [37].

### 4.4. Quantitative PCR for Relative Telomere Length Assessment

Average relative telomere length was measured as previously described [2,28,31,32] with modifications. IFNB1 was used as a single copy gene as published by Vasilishina et al (primer sequences detailed in Table A7). qPCR reactions mixes were prepared according to Table A8 with a standard curve performed for each run. qPCR run in LightCycler 480 II (Roche Diagnostics International Ltd, Rotkreuz, Switzerland) under following conditions: pre-incubation at 95 °C for 10 min, 35 cycles of 95 °C for 15 s, 60 °C for 60 s, and 72 °C for 10 s, followed by melting curve at 95 °C for 5 s, and 65 °C for 60 s. Triplicates for each sample were performed and concentration of sample calculated according to same-run standard curve and averaged for each sample. Relative telomere length (T/S ratio) of each sample was calculated as the ratio between average concentration of telomere reactions to the average concentration of the single copy gene reactions.

### 4.5. Statistical Analyses

All analyses and plots generated and analyzed using JMP 14 (SAS Institute Inc., Cary, NC, USA). For all analyses, *p*-values < 0.05 were considered significant.

## Abbreviations

IMECS	Israeli Multi-Ethnic Centenarian Study
ELLI	Exceptionally Long-Lived Individuals
DNAm	DNA methylation
qPCR	Quantitative Polymerase Chain Reaction
MMSE	Mini-Mental State Exam
CpG	Cytosine Guanine dinucleotide
TL	Telomere Length

## Appendix A

**Table A1:** Age distribution of Israeli Multi-Ethnic Centenarian Study (IMECS) participants.

Group	Number of Participants	Mean Age	STDEV	Min Age	Max Age
**ELLI**	24	97.1	2.04	95	102
**Offspring**	18	70.4	6.25	60	82
**Control**	28	69.7	9.1	53	87

**Table A2:** IMECS demography.

Group	Gender		% Smokers	Education level			
	F	M		Elementary	High School	College Degree	NA
**ELLI**	12	12	57%	4	10	7	3
**Offspring**	12	6	37%	1	3	12	2
**Control**	20	8	39%	0	7	21	0

**Table A3:** One-way analysis of each age measurement through groups.

Age Measure	Group Pairs	Kruskal-Wallis *P*-Value	Wilcoxon Post-Hoc
DNAm PhenoAge	ELLI—Offspring	<0.0001	<0.0001
	ELLI—Control		<0.0001
	Control—Offspring		0.5969
DNAm GrimAge	ELLI—Offspring	<0.0001	<0.0001
	ELLI—Control		<0.0001
	Control—Offspring		0.9731

**Table A4:** Repeated measures test within and among groups.

Measurement Pairs	Within Pairs *P*-Value	Among Pairs *P*-Value
DNAmPhenoAge—actual age	<0.0001	<0.0001
DNAmGrimAge—actual age	<0.0001	<0.0001
DNAmGrimAge—DNAmPhenoAge	0.7812	<0.0001

**Table A5:** Distribution of average T/S ratio.

Group	Median Average T/S	Minimum Average T/S	Maximum Average T/S
**ELLI**	1.01	0.61	5.91
**Offspring**	1.11	0.33	4.23
**Control**	0.95	0.39	2.73

**Table A6:** One-way analysis of DNAmTL measurements.

Group Pairs	ANOVA *P*-Value	Tukey Post-Hoc
ELLI—Offspring	<0.0001	0.0003
ELLI—Control		<0.0001
Control—Offspring		0.3388

**Table A7:** Telomere length qPCR primer information.

Primer Name	Primer Sequence	Working Concentration
Telo-F	CGGTTTGTTTGGGTTTGGGTTTGGGTTTGGGTTTGGGTT	10 µM
Telo-R	GGCTTGCCTTACCCTTACCCTTACCCTTACCCTTACCCT	10 µM
IFNB1-F	GGTTACCTCCGAAACTGAAGA	7.5 µM
IFNB1-R	CCTTTCATATGCAGTACATTAGCC	7.5 µM

**Table A8:** Telomere length qPCR reaction volumes per 1 reaction.

Reagent	Telo Reaction	IFNB1 Reaction
**Ultra-Pure Water (Bio-Lab ltd, Jerusalem, Israel)**	6 µL	4 µL
**SYBR green (Thermo Fisher Scientific Baltics, Vilnius, Lithuania)**	10 µL	10 µL
**F primer (Sigma-Aldrich Israel ltd, Rehovot, Israel)**	0.2 µL	0.6 µL
**R primer (Sigma-Aldrich Israel ltd, Rehovot, Israel)**	1.8 µL	1.4 µL
**Genomic DNA (5 ng/µL)**	2 µL	4 µL

## References

[1] Yashin A., De Benedictis G., Vaupel J., Tan Q., Andreev K., Iachine I., Bonafe M., Valensin S., De Luca M., Carotenuto L. (2000). Genes and longevity: Lessons from studies of centenarians. J. Gerontol. Ser. A Boil. Sci. Med. Sci..

[2] Atzmon G., Cho M., Cawthon R.M., Budagov T., Katz M., Yang X., Siegel G., Bergman A., Huffman D.M., Schechter C.B. (2010). Evolution in health and medicine Sackler colloquium: Genetic variation in human telomerase is associated with telomere length in Ashkenazi centenarians. Proc. Natl. Acad. Sci. USA.

[3] Freudenberg-Hua Y., Freudenberg J., Vacic V., Abhyankar A., Emde A.-K., Ben-Avraham D., Barzilai N., Oschwald D., Christen E., Koppel J. (2014). Disease variants in genomes of 44 centenarians. Mol. Genet. Genom. Med..

[4] Rubino G., Bulati M., Aiello A., Aprile S., Gambino C.M., Gervasi F., Caruso C., Accardi G. (2018). Sicilian centenarian offspring are more resistant to immune ageing. Aging Clin. Exp. Res..

[5] Stevenson M., Bae H., Schupf N., Andersen S., Zhang Q., Perls T., Sebastiani P. (2015). Burden of disease variants in participants of the long life family Study. Aging.

[6] Giuliani C., Sazzini M., Pirazzini C., Bacalini M.G., Marasco E., Ruscone G.A.G., Fang F., Sarno S., Gentilini D., Di Blasio A.M. (2018). Impact of demography and population dynamics on the genetic architecture of human longevity. Aging.

[7] Teixeira L., Araújo L., Jopp D., Ribeiro O. (2017). Centenarians in Europe. Maturitas.

[8] Puca A.A., Spinelli C., Accardi G., Villa F., Caruso C. (2018). Centenarians as a model to discover genetic and epigenetic signatures of healthy ageing. Mech. Ageing Dev..

[9] Milman S., Barzilai N. (2015). Dissecting the Mechanisms Underlying Unusually Successful Human Health Span and Life Span. Cold Spring Harb. Perspect. Med..

[10] Atzmon G., Rincon M., Schechter C.B., Shuldiner A.R., Lipton R.B., Bergman A., Barzilai N. (2006). Lipoprotein genotype and conserved pathway for exceptional longevity in humans. PLoS Boil..

[11] Barzilai N., Atzmon G., Schechter C., Schaefer E.J., Cupples A.L., Lipton R., Cheng S., Shuldiner A.R. (2003). Unique Lipoprotein Phenotype and Genotype Associated With Exceptional Longevity. JAMA.

[12] Ben-Avraham D., Govindaraju D.R., Budagov T., Fradin D., Durda P., Liu B., Ott S., Gutman D., Sharvit L., Kaplan R. (2017). The GH receptor exon 3 deletion is a marker of male-specific exceptional longevity associated with increased GH sensitivity and taller stature. Sci. Adv..

[13] López-Otín C., Blasco M.A., Partridge L., Serrano M., Kroemer G. (2013). The hallmarks of aging. Cell.

[14] Austriaco N.R., Guarente L.P. (1997). Changes of telomere length cause reciprocal changes in the lifespan of mother cells in Saccharomyces cerevisiae. Proc. Natl. Acad. Sci. USA.

[15] Teixeira M.T. (2013). Saccharomyces cerevisiae as a Model to Study Replicative Senescence Triggered by Telomere Shortening. Front. Oncol..

[16] Espejel S., Klatt P., Murcia J.M.-D., Martiín-Caballero J., Flores J.M., Taccioli G., De Murcia G., Blasco M.A. (2004). Impact of telomerase ablation on organismal viability, aging, and tumorigenesis in mice lacking the DNA repair proteins PARP-1, Ku86, or DNA-PKcs. J. Cell Boil..

[17] Honig L.S., Schupf N., Lee J.H., Tang M.X., Mayeux R. (2006). Shorter telomeres are associated with mortality in those with APOE ϵ4 and dementia. Ann. Neurol..

[18] Vera E., De Jesus B.B., Foronda M., Flores J.M., Blasco M.A. (2012). The Rate of Increase of Short Telomeres Predicts Longevity in Mammals. Cell Rep..

[19] Simons M.J. (2015). Questioning causal involvement of telomeres in aging. Ageing Res. Rev..

[20] Harley C.B., Futcher A.B., Greider C.W. (1990). Telomeres shorten during ageing of human fibroblasts. Nature.

[21] Aviv A., Shay J.W. (2018). Reflections on telomere dynamics and ageing-related diseases in humans. Philos. Trans. R. Soc. B Boil. Sci..

[22] Sanders J.L., Newman A.B. (2013). Telomere Length in Epidemiology: A Biomarker of Aging, Age-Related Disease, Both, or Neither?. Epidemiologic Rev..

[23] Xu Z., Duc K.D., Holcman D., Teixeira M.T. (2013). The length of the shortest telomere as the major determinant of the onset of replicative senescence. Genetics.

[24] Savage S.A. (2018). Beginning at the ends: Telomeres and human disease. F1000Research.

[25] Levy D., Neuhausen S.L., Hunt S.C., Kimura M., Hwang S.-J., Chen W., Bis J.C., Fitzpatrick A.L., Smith E., Johnson A.D. (2010). Genome-wide association identifies OBFC1 as a locus involved in human leukocyte telomere biology. Proc. Natl. Acad. Sci. USA.

[26] Terry D.F., Nolan V.G., Andersen S.L., Perls T.T., Cawthon R. (2008). Association of longer telomeres with better health in centenarians. Journals Gerontol. Ser. A: Boil. Sci. Med Sci..

[27] Gutman D., Sharvit L., Atzmon G. (2014). Possible Mechanisms for Telomere Length Maintenance in Extremely Old People. Hered. Genet..

[28] Cawthon R.M. (2002). Telomere measurement by quantitative PCR. Nucleic Acids Res..

[29] Pfaffl M.W. (2001). A new mathematical model for relative quantification in real-time RT-PCR. Nucleic Acids Res..

[30] Cawthon R.M., Smith K.R., O’Brien E., Sivatchenko A., Kerber R.A. (2003). Association between telomere length in blood and mortality in people aged 60 years or older. Lancet.

[31] Shekhidem H.A., Sharvit L., Leman E., Manov I., Roichman A., Holtze S., Huffman D.M., Cohen H.Y., Hildebrandt T.B., Shams I. (2019). Telomeres and Longevity: A Cause or an Effect?. Int. J. Mol. Sci..

[32] Vasilishina A., Kropotov A., Spivak I., Bernadotte A. (2019). Relative human telomere length quantification by real-time PCR. Methods Mol. Biol..

[33] Axelrad M.D., Budagov T., Atzmon G. (2013). Telomere Length and Telomerase Activity; A Yin and Yang of Cell Senescence. J. Vis. Exp..

[34] Montpetit A.J., Alhareeri A.A., Montpetit M., Starkweather A.R., Elmore L.W., Filler K., Mohanraj L., Burton C.W., Menzies V.S., Lyon D.E. (2014). Telomere length: A review of methods for measurement. Nurs. Res..

[35] Hannum G., Guinney J., Zhao L., Zhang L., Hughes G., Sadda S., Klotzle B., Bibikova M., Fan J.-B., Gao Y. (2013). Genome-wide methylation profiles reveal quantitative views of human aging rates. Mol. Cell.

[36] Horvath S., Pirazzini C., Bacalini M.G., Gentilini D., Di Blasio A.M., Delledonne M., Mari D., Arosio B., Monti D., Passarino G. (2015). Decreased epigenetic age of PBMCs from Italian semi-supercentenarians and their offspring. Aging.

[37] Horvath S. (2013). DNA methylation age of human tissues and cell types. Genome Boil..

[38] Chen B.H., Marioni R.E., Colicino E., Peters M.J., Ward-Caviness C.K., Tsai P.-C., Roetker N.S., Just A.C., Demerath E.W., Guan W. (2016). DNA methylation-based measures of biological age: Meta-analysis predicting time to death. Aging.

[39] Horvath S., Oshima J., Martin G.M., Lu A.T., Quach A., Cohen H., Felton S., Matsuyama M., Lowe D., Kabacik S. (2018). Epigenetic clock for skin and blood cells applied to Hutchinson Gilford Progeria Syndrome and ex vivo studies. Aging.

[40] Armstrong N.J., Mather K.A., Thalamuthu A., Wright M.J., Trollor J.N., Ames D., Brodaty H., Schofield P.R., Sachdev P.S., Kwok J.B. (2017). Aging, exceptional longevity and comparisons of the Hannum and Horvath epigenetic clocks. Epigenomics.

[41] Ryan J., Wrigglesworth J., Loong J., Fransquet P.D., Woods R.L., Anderson R. (2019). A systematic review and meta-analysis of environmental, lifestyle and health factors associated with DNA methylation age. J. Gerontol. Ser. A Boil. Sci. Med. Sci..

[42] Lu A.T., Quach A., Wilson J.G., Reiner A.P., Aviv A., Raj K., Hou L., Baccarelli A.A., Li Y., Stewart J.D. (2019). DNA methylation GrimAge strongly predicts lifespan and healthspan. Aging.

[43] Lu A.T., Seeboth A., Tsai P.-C., Sun D., Quach A., Reiner A.P., Kooperberg C., Ferrucci L., Hou L., Baccarelli A.A. (2019). DNA methylation-based estimator of telomere length. Aging.

[44] Levine M.E., Lu A.T., Quach A., Chen B.H., Assimes T.L., Bandinelli S., Hou L., Baccarelli A.A., Stewart J.D., Li Y. (2018). An epigenetic biomarker of aging for lifespan and healthspan. Aging.

[45] Fahy G.M., Brooke R.T., Watson J.P., Good Z., Vasanawala S.S., Maecker H., Leipold M.D., Lin D.T.S., Kobor M.S., Horvath S. (2019). Reversal of epigenetic aging and immunosenescent trends in humans. Aging Cell.

[46] Xiao F.-H., Wang H.-T., Kong Q.-P. (2019). Dynamic DNA Methylation During Aging: A “Prophet” of Age-Related Outcomes. Front. Genet..

[47] Bernadotte A., Mikhelson V.M., Spivak I.M. (2016). Markers of cellular senescence. Telomere shortening as a marker of cellular senescence. Aging.

[48] Lin Q., Weidner C.I., Costa I.G., Marioni R.E., Ferreira M.R.P., Deary I.J., Wagner W. (2016). DNA methylation levels at individual age-associated CpG sites can be indicative for life expectancy. Aging.

[49] Shiels P.G., Stenvinkel P., Kooman J.P., McGuinness D. (2017). Circulating markers of ageing and allostatic load: A slow train coming. Pract. Lab. Med..

[50] Olivieri F., Capri M., Bonafè M., Morsiani C., Jung H.J., Spazzafumo L., Viña J., Suh Y. (2017). Circulating miRNAs and miRNA shuttles as biomarkers: Perspective trajectories of healthy and unhealthy aging. Mech. Ageing Dev..

[51] Deelen J., Akker E.B.V.D., Trompet S., Van Heemst D., Mooijaart S.P., Slagboom P.E., Beekman M. (2016). Employing biomarkers of healthy ageing for leveraging genetic studies into human longevity. Exp. Gerontol..

[52] Barral S., Singh J., Fagan E., Cosentino S., Andersen-Toomey S.L., Wojczynski M.K., Feitosa M., Kammerer C.M., Schupf N., Long Life Family Study (2017). Age-Related Biomarkers in LLFS Families With Exceptional Cognitive Abilities. J. Gerontol. Ser. A Boil. Sci. Med Sci..

[53] Aryee M.J., Jaffe A.E., Corrada-Bravo H., Ladd-Acosta C., Feinberg A.P., Hansen K.D., Irizarry R.A. (2014). Minfi: A flexible and comprehensive Bioconductor package for the analysis of Infinium DNA methylation microarrays. Bioinformatics.

[54] Jaffe A.E., Murakami P., Lee H., Leek J.T., Fallin M.D., Feinberg A.P., Irizarry R.A. (2012). Bump hunting to identify differentially methylated regions in epigenetic epidemiology studies. Int. J. Epidemiol..

